# Medication Discrepancies in Community Pharmacies in Switzerland: Identification, Classification, and Their Potential Clinical and Economic Impact

**DOI:** 10.3390/pharmacy8010036

**Published:** 2020-03-09

**Authors:** Tamara L Imfeld-Isenegger, Melanie Bich Tram Pham, Dominik Stämpfli, Valerie Albert, Enas Almanasreh, Rebekah Moles, Timothy F Chen, Kurt E Hersberger

**Affiliations:** 1 Pharmaceutical Care Research Group, University of Basel, 4056 Basel, Switzerland; melanie.pham94@gmail.com (M.B.T.P.); valerie.albert@unibas.ch (V.A.); kurt.hersberger@unibas.ch (K.E.H.); 2 Pharmacoepidemiology, Institute of Pharmaceutical Sciences, Department of Chemistry and Applied Biosciences, ETH Zurich, 8093 Zurich, Switzerland; dominik.staempfli@pharma.ethz.ch; 3 Faculty of Pharmacy, Pharmacy and Bank Building A15, The University of Sydney, Camperdown NSW 2006, Australia; almanasreh.enas@gmail.com (E.A.); rebekah.moles@sydney.edu.au (R.M.); timothy.chen@sydney.edu.au (T.F.C.)

**Keywords:** medication discrepancy, medication reconciliation, medication review, community pharmacy, pharmaceutical care

## Abstract

**Background**: Transitions of care are high-risk situations for the manifestation of medication discrepancies and, therefore, present threats for potential patient harm. Medication discrepancies can occur at any transition within the healthcare system. **Methods**: Fifth-year pharmacy students assessed a best possible medication list (BPML) during a medication review (based on medication history and patient interview) in community pharmacies. They documented all discrepancies between the BPML and the latest medication prescription. Discrepancies were classified using the medication discrepancy taxonomy (MedTax) classification system and were assessed for their potential clinical and economic impact. **Results:** Overall, 116 patients with a mean age and medication prescription of 74 (± 10.3) years and 10.2 (± 4.2), respectively, were analyzed. Of the 317 discrepancies identified, the most frequent type was related to strength and/or frequency and/or number of units of dosage form and/or the total daily dose. Although, the majority of discrepancies were rated as inconsequential (55.2%) on health conditions, the remainder posed a potential moderate (43.2%) or severe impact (1.6%). In 49.5% of the discrepancies, the current patients’ medication cost less than the prescribed. **Conclusion**: Community pharmacies are at a favorable place to identify discrepancies and to counsel patients. To improve patient care, they should systematically perform medication reconciliation whenever prescriptions are renewed or added.

## 1. Introduction

Transitions of care, especially hospital admission and discharge, are high-risk situations for the manifestation of medication discrepancies and therefore relevant origins for potential patient harm [1,2,3]. Medication discrepancy is defined as “any differences in the prescribed medication, dose, route, or frequency noted among the sources of documentation.” [4]. An intentional medication discrepancy is when prescribers intentionally decide to add, modify, or remove a patient’s medication [5]. Unintentional discrepancies occur when prescribers unintentionally add, change, or remove medications from the medication list [5]. The latter type of medication discrepancies can lead to adverse drug events [5].

At hospital admission, the number of medication discrepancies per patient ranges from 3.0 to 9.8 [6,7,8], whereas at hospital discharge, the average ranges from 0.6 to 4.0 [9,10,11] discrepancies per patient. Pharmacists play a pivotal role in the medication reconciliation process, because they are able to detect and resolve medication discrepancies [3]. Medication reconciliation as defined by the Medical Subject Headings (MeSH) is a “formal process of obtaining a complete and accurate list of each patient’s current home medications including name, dosage, frequency, and route of administration, and comparing admission, transfer, and/or discharge medication orders to that list. The reconciliation is done to avoid medication errors.” Multiple studies focusing on medication reconciliation at hospital transitions were published [2,3,12,13]. The meta-analysis by Mekonnen and colleagues [2] indicated a reduction in emergency department visits and hospital readmission in patients with a pharmacist-led medication reconciliation procedure at hospital transitions. However, medication discrepancies can also occur outside of hospital admission and discharge at any transition in the healthcare system, e.g., at transitions from medical specialists, general practitioners, community pharmacies, and nursing homes, or even without any transition [14,15,16].

The aim of this study was to identify, characterize, and categorize medication discrepancies occurring in adult community pharmacy customers with long-term polypharmacy use and to assess their potential clinical and economic impact.

## 2. Materials and Methods

The study presented here was part of a larger cross-sectional observational study across Swiss community pharmacies with three different investigated topics: (1) medication management (investigation of patients’ organization strategies with their polypharmacy in daily life), (2) attitudes towards generic medication, and (3) medication discrepancies between current patient medication and the latest medication prescription filled in the community pharmacy in patients with polypharmacy (≥4 medications) for long-term use (>3 months). In this study, we analyzed part (3) on medication discrepancies. All patients and community pharmacies gave written informed consent for study participation. The study was conducted in accordance with the Declaration of Helsinki, and was approved by the Northwest and Central Switzerland ethics committee (EKNZ 2016-02143) and registered on ClinicalTrials.gov (NCT03321058).

### 2.1. Setting and Recruitment

**Figure 1:**
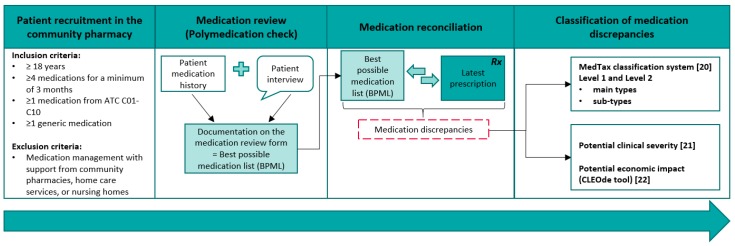
Study flowchart, ATC = Anatomical Therapeutic Chemical; BPML = best possible medication list; Rx = medication prescription; MedTax = medication discrepancy taxonomy [20]; CLEOde tool = evaluation of the potential CLinical, Economic, and Organizational relevance of pharmacists’ interventions, German version [22].

Two consecutive cohorts of 5th year pharmacy students of the University of Basel, working in Swiss community pharmacies during their internship, served as recruiters for this study. The number of patients included in this study was given by the number of students in the two student cohorts. Each student attended one medication review in the community pharmacy and performed a medication reconciliation (Figure 1). For this medication review, students selected a regular patient from the community pharmacy in accordance with the inclusion and exclusion criteria. Eligibility criteria were adult patient (≥18 years) with ≥4 medications for a minimum of 3 months, including ≥1 medication from the Anatomical Therapeutic Chemical (ATC) group cardiovascular system (C01–C10), and ≥1 generic medication. Exclusion criteria were managing their medication with professional support from pharmacies, home care services, or nursing homes. These inclusion and exclusion criteria were used to investigate the medication management, the attitudes towards generic medication, and for the assessment of medication discrepancies. 

### 2.2. Medication Review

The pharmacist-led medication reviews, called “Polymedication Checks” (PMC) in Switzerland [17], were performed by the supervisors of the students, but in presence of the students. According to the classification of the Pharmaceutical Care Network Europe [18], the PMC is a type 2a medication review based on a routinely collected patient medication history and an additional patient interview [17]. The documentation of dispensed medication in patient medication histories are required for all patients filling prescriptions in community pharmacies in Switzerland. Medication name, strength, dosage, time of administration, and indication are documented on the standardized medication review form. The PMC focuses on medication use, patient knowledge, and medication adherence, and results in a list of the patient’s current home medication. The PMC is an implemented and remunerated cognitive community pharmacy service in Switzerland [19]. Pharmacy students were trained to perform medication reviews during lectures and workshops at the university. Prior to the start of the study, all students additionally received a verbal introduction and a written manual for performing medication reviews.

### 2.3. Medication Reconciliation

**Figure A1:**
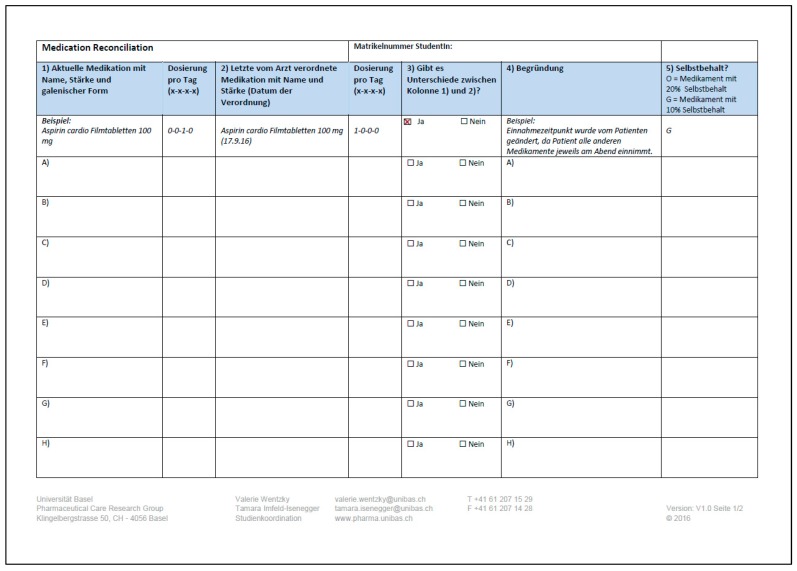
Medication reconciliation form.

The list of the patient’s prescribed home medication assessed during the PMC (based on the patient medication history + patient interview) was used as best possible medication list (BPML) in this study (Figure 1). The students then transferred this BPML to a separate, standardized medication reconciliation form with two columns: one for the BPML and the other for the medication according to the latest medication prescription available in the community pharmacy (Figure A1). They compared each medication and marked any discrepancy. In case of a discrepancy (intentional or unintentional), they added a description and/or potential explanation of the discrepancy as free text.

### 2.4. Data Handling

The medication review form, the patient’s medication history with the comprehensive refill data for a period of 12-months, and the medication reconciliation form were pseudonymized and sent to the study site, where the results were entered into a database. The ATC codes were amended to all active ingredients involved in a medication discrepancy. Subsequently, all discrepancies were categorized with medication discrepancy taxonomy (MedTax) [20], a validated classification system for medication discrepancies. This taxonomy is hierarchical and consists of two levels (1 “medication mismatched”, 2 “medication partially matched”), with 12 main types and 28 sub-types [20]. For level 1 “medication mismatched”, the active substance of the medication was either present on the BPML or on the latest medication prescription. In contrast, in level 2 “medication partially matched”, the active substance was identical on BPML and the latest medication prescription.

The plausibility of the discrepancies reported by the students were checked using their free text comments, the medication review form, and the 12-months medication history. This plausibility check and the categorization of the medication discrepancies were independently performed by two researchers (T.L.I.-I., M.B.T.P.) and subsequently discussed in a meeting. In case of disagreement, a third person (K.E.H.) joined the discussion.

### 2.5. Potential Clinical Severity and Economic Impact of the Identified Medication Discrepancies

Three clinical pharmacists with working experience in hospital pharmacies and community pharmacies (V.A., D.S., T.L.I.-I.) assessed the potential clinical severity and economic impact of all medication discrepancies. Any disagreement among the three pharmacists were individually re-evaluated. In case of further disagreement, the majority decided. They evaluated the potential direct or indirect clinical impact of each medication discrepancy on the patient’s medical conditions using three different severity classes: “unlikely” (Class 1), “moderate” (Class 2), or “potentially severe” (Class 3), as adapted from Cornish and colleagues [21]. The discrepancy, hence, either leads to a potential positive or potential negative effect on the patient’s health condition in context of the entire medication.

The potential economic impact of the medication discrepancy was assessed using the economic dimension of the tool CLEOde (CLinical, Economic, and Organizational), a validated tool for the assessment of the potential relevance of pharmacists’ interventions [22]. In this study, the economic dimension of CLEOde was defined as immediate impact of medication discrepancies on the medication costs (levels: increase, null, decrease) from a healthcare system’s perspective by comparing the medication of the BPML and the latest medication prescription.

### 2.6. Statistical Analysis

Demographic patient data, number of medications and related ATC codes, number of generic medications, and frequency and characteristics of medication discrepancies were analyzed. Data were descriptively quantified and analyzed using IBM^®^ SPSS^®^ Statistics Version 25 (IBM Corp., Armonk, NY, USA). The inter-rater reliability for the assessment of the potential clinical and economic impact of the medication discrepancies was analyzed using Fleiss’ Kappa [23] from IBM^®^ SPSS^®^ and interpreted according to Landis and Koch [24].

## 3. Results

A total of 149 pharmacy students attended medication reviews during their practical training in the community pharmacy between January and August 2017 in Switzerland. Six community pharmacies and two patients denied study participation; three patients did not meet the inclusion criteria, while six were excluded by the research team due to missing or invaluable data (e.g., inconsistent information). Sixteen patients were excluded, because they obtained their prescribed medications in the physician’s practice or filled the prescriptions in another community pharmacy. Therefore, the medication history in the participating community pharmacy was inadequate for the BPML. In total, 116 medication reconciliation forms originating from 96 different community pharmacies were included for analysis (20 community pharmacies with two students). The mean age of the patients was 74 (± 10.3) years (range 49 to 91 years). Gender was male for 56.9% (66/116) of the patients. Overall, 79.3% of the study patients were ≥65 years old. Patients had, on average, 10.2 ± 4.2 medications (range 4 to 26) on the BPML, including at least one medication from the ATC group C (cardiovascular) and at least one generic medication.

### 3.1. Medication Discrepancies—Frequency of Main Types

In total, the students identified 317 medication discrepancies in 116 patients, with a mean of 2.7 ± 2.3 discrepancies per patient (range 0–11). In 82.8% (96/116) of all patients, at least one medication discrepancy was detected. A total of 1180 prescribed medications were listed on 116 different medication review forms. Of the 317 medication discrepancies, one-third (34.1%) were medications from the ATC group C for the cardiovascular system, while 25.2% medications were from the ATC group A for the alimentary tract and metabolism, and 13.9% from the group N for the nervous system (Table 1).

Table 2 shows the two levels of the MedTax classification system [20] with the according 12 main types of medication discrepancies. Over 90% (n = 299) of the discrepancies were categorized as level 2 “medication partially mismatched”.

The most frequently identified main type of medication discrepancy (42.3%) was “2.2 discrepancy in the strength and/or frequency and/or number of units of dosage form and/or total daily dose”. The main type “2.6 other” was the second most often identified type of discrepancy (e.g., stop of a medication by the physician with knowledge of the patient, but without transfer of this information to the community pharmacy). An additional, frequently observed discrepancy (13.6%) was “2.1 discrepancy in the name of medication” caused by different medication names (brand to generic name, generic to brand name, and generic to generic name).

### 3.2. Medication Discrepancies—Frequency of Sub-Types

Level 2 “medication partially mismatched” discrepancies (categories 2.1–2.4) were further specified to different sub-types according to the MedTax [20] classification system (Table 3). The most frequent sub-type in the category “2.2 discrepancy in the strength and/or frequency and/or number of units of dosage form and/or total daily dose” was “2.2.7 same strength but unclear or wrong frequency” caused by the prescription of an on-demand regimen (pro re nata; PRN), but the patient took the medication on a regular basis or vice versa. One reason for the discrepancy in the sub-type “2.4 discrepancy in the time of medication administration” was that medication was administered at different times through the day to improve adherence or effectiveness of a medication.

### 3.3. Potential Clinical Severity of the Medication Discrepancies and Economic Impact

The potential clinical severity of the medication discrepancies and the economic impact are presented in Table 4. Three clinical pharmacists assessed and categorized the potential clinical severity and economic impact of the medication discrepancies. Fleiss’ kappa coefficient was moderate for the rating of the potential clinical severity (κ = 0.478, *p* < 0.0005) and substantial for the potential economic impact (κ = 0.637, *p* < 0.0005). Over half (55.2%) of the identified medication discrepancies were considered as unlikely to have a clinical effect on the patient’s health condition (Class 1), whereas 1.6% were rated to have a potentially severe impact on the patient’s health condition (Class 3), either in a positive or negative sense. The following five medication discrepancies were rated as a potentially severe clinical impact: 1) a patient accidentally took rivaroxaban 20 mg twice daily instead of once daily as prescribed by the physician; 2) unclear dose of methotrexate; 3) stop of methotrexate; 4) use of indacaterol for acute congested bronchi; and 5) an intentional change in the dosing of a rapid-acting insulin (from 11 IU to 30–50 IU per day).

In 157 of the 317 medication discrepancies (49.5%), the difference between the prescription and the current patient medication led to a decrease in medication therapy costs (e.g., reduction of the dose or frequency, stop of a medication, generic substitution). In contrast, 49 (15.5%) of the medication discrepancies caused an increase in costs (e.g., increase in the total daily dose, therapeutic class substitution, regular intake of an as-needed medication).

## 4. Discussion

In this study, pharmacy students performed medication reconciliation in community pharmacies in patients with polypharmacy for long-term use, based on a BPML compiled through a type 2a medication review in Swiss community pharmacies. The population consisted of elderly patients (mean age 74 years) with an average of 10.2 medications at the time of the medication review. In total, 317 medication discrepancies were detected between the BPML and the latest prescription of the 116 patients (2.7 medication discrepancies/patient), and 82.8% of patients had a minimum of one medication discrepancy. Some of the identified discrepancies were intentional changes by the patient (e.g., different intake time during the day) and some were unintentional discrepancies (e.g., mix-up or overdose of a product). Our finding of 2.7 discrepancies per patient is consistent with two other studies performed in the primary care setting [14,15]. Rose and colleagues [15] compared patient reported medication lists assessed during a home visit to physician records and they identified at least one medication discrepancy in 94.4% of the patients. They found 2.8 ± 2.4 medication discrepancies per patient [15]. This average number of discrepancies was slightly lower than the results from Andrus and colleagues [14] with 3.2 discrepancies per patient, investigating the actual patient medication with the electronic health record in an outpatient family clinic in the United States.

### 4.1. Types of Medication Discrepancies

The three types of discrepancies that occurred most frequently in our study were (1) discrepancy in the strength/frequency/number of units/total daily dose, (2) discrepancy in the name of the medication, and (3) other. These types of discrepancies belong to level 2 of the MedTax classification system (partially mismatched medications) [20]. In this level, the active ingredient of the medication with a discrepancy was identical on both lists. It is not surprising that only few discrepancies (5.7%) in our study were discrepancies within level 1 “medication mismatched”, because the start or stop of medications leading to commission or omission are in the responsibility of the physician in Switzerland. In contrast, studies performed at hospital admissions or discharges revealed medication mismatches, such as omission, commission, and therapeutic class substitution, more frequently [10,11,25,26]. The omission of a medication was the most frequently reported discrepancy after hospital admission or discharge [11,21,25,26]. Similarly, Almanasreh and colleagues [27] identified omission as the most frequent type of discrepancy in their systematic review focusing on the medication reconciliation process and medication discrepancy classification. However, it should be considered that the lack of a standardized classification system led to various pragmatically developed classifications for medication discrepancies. This heterogeneity in turn makes comparisons of different studies difficult [27].

The sub-type of medication discrepancy that occurred most frequently in our study was a difference in the name of the medication. This included differences between brand and generic names, as well as between two products from different generics manufacturers. Even though the active ingredient remains unchanged, any generic substitution may lead to a change in the primary packaging, the appearance of the blister and the shape, as well as the size or color of the dosage form. These differences are important issues for patients, because they may lead to confusion, non-adherence [28], and a perceived altered effect of the medication [29]. Sufficient information by pharmacists or physicians about the generic substitution may reduce the patient’s feeling of insecurity [28] or dissatisfaction [30]. Community pharmacists in Switzerland are allowed to substitute the prescribed medication by a product from another manufacturer, unless the prescriber restricts it [31]. To avoid patients’ concerns, confusion, and potential non-adherence, pharmacists might dispense the medication known by the patient even though another medication product was prescribed by the physician. This substitution results in an intentional discrepancy. 

Another frequent sub-type was medication with the same strength, but unclear or wrong frequency of administration. An example of this type of discrepancy was that patients often took their medication on a demand regimen (PRN medication) rather than on a scheduled regimen as prescribed by the physician, or vice versa. The failure of the patients to comply with the prescribed dosing regimen is well known and is, therefore, a frequent reason for discrepancies observed in ambulatory care [14]. Some of the medication discrepancies occurred due to intentional changes by the patients themselves (e.g., change of the intake time) or by the physicians (e.g., verbal instructions to patients to change dose, or to intentionally stop a medication). According to annotations by the pharmacists and the pharmacy students in the medication review forms, a frequently reported discrepancy was an intentional stop of a medication by the physician with the knowledge of the patient, but without the transfer of this information to the community pharmacy. Subsequently, patients reported this intentional stop to the community pharmacist during the medication review. The lack of communication between community pharmacists and physicians create issues with care transitions and was identified as a potential risk for patient safety [32,33]. Eggink and colleagues [34] reported that a clinical pharmacist-led discharge service consisting of a medication review, verbal and written information for the patient, and a discharge list with additional information related to medication, decreased the number of prescription errors and medication discrepancies in patients with heart failure. Furthermore, in a survey in the UK, 89% of general practitioners and 76% of community pharmacists wished to have additional information, such as discontinuation of a medication [35]. This lack of information within the community pharmacy about intentional or unintentional medication discontinuation or dose regimen changes are a risk for patients and needs time to be solved by healthcare professionals in primary care [36]. A pragmatic pharmacist-led in-hospital service consisting of medication reconciliation, addition of information regarding therapy changes (new, stop, change in dose), and check of the prescription for formal mistakes reduced clinically significant pharmaceutical interventions. Moreover, pharmacists’ satisfaction with the quality of the discharge prescription were significantly higher in the group with the in-hospital service [37]. The performance of a systematic medication reconciliation as part of a medication review in community pharmacies is also a chance to update the community pharmacy patient records and the patient’s home medication list. A Dutch study reported poor documentation of medication changes after hospital discharge in the patient records in the community pharmacy, which could lead to a negative effect on the continuity of care [38].

### 4.2. Potential Clinical Severity and Economic Impact

Focusing on the potential clinical effects of the detected medication discrepancies, only 1.6% of the medication discrepancies were rated as having a potentially severe effect and 43.2% were assessed as having a potentially moderate clinical effect on patients’ health conditions. Over half (55.2%) of the medication discrepancies were rated to be unlikely to have an effect. This pattern of potential severity is consistent with the results from studies by Cornish et al. [21] and Becerra-Camargo et al. [25] investigating unintentional medication discrepancies at hospital discharge and admission, respectively. Although the potentially clinically severe discrepancies accounted for a small proportion of the discrepancies in these studies, all kinds of medication discrepancies have a potential risk for adverse drug events and should, therefore, be identified and resolved. Pharmacist-led interventions consisting of a systematic medication reconciliation and periodic medication reviews could help to identify, address, and resolve these unfavorable medication discrepancies, and could, therefore, reduce drug-related problems. Focusing on the economic effect of medication reconciliation, Kennelty et al. [39] identified barriers and facilitators of medication reconciliation after discharge by interviewing 10 community pharmacists. They reported that reduced costs due to a decrease of unnecessary healthcare utilization by preventing medication errors were benefits of medication reconciliation after discharge. Our study analyzed the potential immediate economic impact of medication discrepancies on the current medication therapy costs. In approximately 50% (n = 157) of the discrepancies, the medication therapy costs were lower than the therapy initially prescribed by the physician. This result is mainly based on discontinued medications, dose reductions, or generic medication substitutions, which all lower the immediate and actual drug use costs.

### 4.3. Strengths and Limitations

This study was performed under real-world conditions in various community pharmacies across Switzerland. The PMC demonstrated to be a useful tool to compile a BMPL and identify intentional and unintentional changes in patients’ medication in the community pharmacy. We acknowledge few limitations. First, the uncontrolled patient recruitment and the inclusion criteria (including >1 medication from the ATC group cardiovascular system and >1 generic medication) poses a selection bias, which may influence the frequency of medication discrepancies and medication classes and limits the generalizability of these results. Nevertheless, the pattern of medication classes is comparable to previous studies [21,25,40,41]. Second, the clinical and economic impacts were retrospectively assessed based on the aggregated information and short descriptions handed in by the students. We deemed the aggregated information (PMC, medication history over 12 months, medication reconciliation form) and short descriptions as correct and sufficient to allow a retrospective evaluation of the medication discrepancies. Moreover, we had a good inter-rater reliability for the potential clinical severity (moderate) and economic impact (substantial). Third, the assessment of the absolute costs was generated by, e.g., missed doses, and did not include therapeutic failure or clinical outcomes.

## 5. Conclusions

Medication reconciliation processes in community pharmacies lead to the identification of numerous medication discrepancies between the patients’ current home medication and the latest medication prescription. Most discrepancies were classified as partially mismatched medication, whilst omission, duplication, and commission accounted for less than 10% of all discrepancies, which is typical for the ambulatory setting. The results of this study show that within the healthcare system, community pharmacies are favorably placed to perform medication reconciliation and to counsel patients on discrepancies. To improve patient care, community pharmacists should systematically perform medication reconciliation prior to a medication review and whenever prescriptions are renewed or added.

## Figures and Tables

**Table 1:** Frequency of the ATC main group causing medication discrepancies (n = 317).

ATC Group	Contents	Number (%)
A	Alimentary Tract and Metabolism	80 (25.2)
B	Blood and Blood Forming Organs	25 (7.9)
C	Cardiovascular System	108 (34.1)
D	Dermatologicals	6 (1.9)
G	Genito Urinary System and Sex Hormones	3 (0.9)
H	Systemic Hormonal Preparations, Excl. Sex Hormones and Insulines	7 (2.2)
J	Antiinfectives for Systemic Use	1 (0.3)
L	Antineoplastic and Immunomodulating Agents	3 (0.9)
M	Musculo-Skeletal System	24 (7.6)
N	Nervous System	44 (13.9)
P	Antiparasitic Products, Insecticides, and Repellents	2 (0.6)
R	Respiratory System	11 (3.5)
S	Sensory Organs	3 (0.9)
V	Various	0 (0.0)

**Table 2:** Classification of medication discrepancies using the MedTax classification system [20]—main types (n = 317).

Levels and Main Types	Number (%)
1. Medication mismatched	18 (5.7)
1.1 Medication omission	0 (0.0)
1.2 Medication commission (or addition)	2 (0.6)
1.3 Medication duplication	1 (0.3)
1.4 Therapeutic class substitution (medication change within a medication class)	12 (3.8)
1.5 Allergy or intolerance*	0 (0.0)
1.6 Other	3 (0.9)
2. Medication partially matched	299 (94.3)
2.1 Discrepancy in the name of medication	43 (13.6)
2.2 Discrepancy in the strength and/or frequency and/or number of units of dosage form and/or total daily dose	134 (42.3)
2.3 Discrepancy in the dosage form/route of administration	0 (0.0)
2.4 Discrepancy in the time of medication administration	35 (11.0)
2.5 Discrepancy in the duration or length of the therapy	0 (0.0)
2.6 Other	87 (27.4)

* Allergies and intolerances were not assessed during the medication review.

**Table 3:** Classification of medication discrepancies using the MedTax classification system [20] focussing on partially matched medication (n = 299) and their classification in the sub-types (n = 212 sub-types).

Main Types and Sub-Types	Number (%)
**2.1. Discrepancy in the name of medication**	**43 (13.6)**
2.1.1. Unclear or wrong name (brand name or generic name)	-
2.1.2. Omission of brand name	-
2.1.3. Omission of generic name	-
2.1.4 Different brand name but same generic name	43 (13.6)
**2.2. Discrepancy in the strength and/or frequency and/or number of units of dosage form and/or total daily dose**	**134 (42.3)**
2.2.1. Unclear or wrong strength	-
2.2.2. Omission of strength	-
2.2.3. Different strength and different total daily dose	8 (2.5)
2.2.4. Different strength but same total daily dose	4 (1.3)
2.2.5. Omission of unit of strength	-
2.2.6. Different or wrong unit of strength	-
2.2.7. Same strength but unclear or wrong frequency	40 (12.6)
2.2.8. Same strength but omission of frequency	29 (9.1)
2.2.9. Same strength but different frequency and omission of the number of units	-
2.2.10. Same strength and same frequency but omission of the number of units	-
2.2.11. Same strength and same number of units but different frequency and different total daily dose	21 (6.6)
2.2.12. Same strength but different frequency and different number of units and different total daily dose	9 (2.8)
2.2.13. Same strength but different frequency and different number of units but same total daily dose	4 (1.3)
2.2.14. Same strength and same frequency but different number of units and different total daily dose	19 (6.0)
**2.3 Discrepancy in the dosage form / route of administration**	**0 (0.0)**
2.3.1. Unclear or wrong dosage form	-
2.3.2. Unclear or wrong route of administration	-
2.3.3. Omission of dosage form	-
2.3.4. Omission of route of administration	-
2.3.5. Different dosage form but same route of administration	-
2.3.6. Different dosage form and different route of administration	-
2.3.7. Same dosage form but different route of administration	-
**2.4. Discrepancy in the time of medication administration**	**35 (11.0)**
2.4.1. Omission of the time of administration	3 (0.9)
2.4.2. Different time of administration through the day	25 (7.9)
2.4.3. Discrepancy in the medication administration with respect to food/meal	7 (2.2)
**2.5. Discrepancy in the duration or length of therapy**	**0 (0.0)**
**2.6. Other**	**87 (27.4)**

**Table 4:** Type of medication discrepancy, its potential clinical severity, and economic impact (n = 317) indicated with numbers (%).

Medication Discrepancy		Potential Clinical Severity ^a^			Potential Economic Impact ^b^		
Type	No. (%)	Class 1*	Class 2*	Class 3*	Increase in Cost	Null	Decrease in Cost
1.1 Medication omission	-	-	-	-	-	-	-
1.2 Medication commission	2 (0.6)	-	2 (0.6)	-	2 (0.6)	-	-
1.3 Medication duplication	1 (0.3)	-	1 (0.3)	-	1 (0.3)	-	-
1.4 Therapeutic class substitution	12 (3.8)	2 (0.6)	10 (3.2)	-	3 (0.9)	1 (0.3)	8 (2.5)
1.5 Allergy or intolerance*	-	-	-	-	-	-	-
1.6 Other (mismatched)	3 (0.9)	1 (0.3)	2 (0.6)	-	3 (0.9)	-	-
2.1 Discrepancy in the name of the medication	43 (13.6)	42 (13.2)	1 (0.3)	-	3 (0.9)	23 (7.3)	17 (5.4)
2.2 Discrepancy in the strength and/or frequency and/or number of units of dosage form and/or total daily dose	134 (42.3)	60 (18.9)	71 (22.4)	3 (0.9)	29 (9.1)	43 (13.6)	62 (19.6)
2.3 Discrepancy in the dosage form/route of administration	-	-	-	-	-	-	-
2.4 Discrepancy in the time of medication administration	35 (11.0)	31 (9.8)	4 (1.3)	-	-	35 (11.0)	-
2.5 Discrepancy in the duration or length of therapy	-	-	-	-	-	-	-
2.6 Other (partially matched)	87 (27.4)	39 (12.3)	46 (14.5)	2 (0.6)	8 (2.5)	9 (2.8)	70 (22.1)
Total	317 (100.0)	175 (55.2)	137 (43.2)	5 (1.6)	49 (15.5)	111 (35.0)	157 (49.5)

^a^ Classification adapted from Cornish et al. [21]. ^b^ Classification adapted from Stämpfli et al. [22]. Class 1 = unlikely to have an effect, Class 2 = potential moderate effect, Class 3 = potential severe effect. * Allergies and intolerances were not assessed during the medication review.
